# An ensemble machine learning model generates a focused screening library for the identification of CDK8 inhibitors

**DOI:** 10.1002/pro.5007

**Published:** 2024-05-09

**Authors:** Tony Eight Lin, Dyan Yen, Wei‐Chun HuangFu, Yi‐Wen Wu, Jui‐Yi Hsu, Shih‐Chung Yen, Tzu‐Ying Sung, Jui‐Hua Hsieh, Shiow‐Lin Pan, Chia‐Ron Yang, Wei‐Jan Huang, Kai‐Cheng Hsu

**Affiliations:** ^1^ Graduate Institute of Cancer Biology and Drug Discovery, College of Medical Science and Technology Taipei Medical University Taipei Taiwan; ^2^ Ph.D. Program for Cancer Molecular Biology and Drug Discovery College of Medical Science and Technology, Taipei Medical University Taipei Taiwan; ^3^ TMU Research Center of Cancer Translational Medicine Taipei Medical University Taipei Taiwan; ^4^ Warshel Institute for Computational Biology The Chinese University of Hong Kong (Shenzhen) Shenzhen Guangdong People's Republic of China; ^5^ Biomedical Translation Research Center, Academia Sinica Taipei Taiwan; ^6^ Division of Translational Toxicology National Institute of Environmental Health Sciences, National Institutes of Health Durham North Carolina USA; ^7^ School of Pharmacy, College of Medicine National Taiwan University Taipei Taiwan; ^8^ Graduate Institute of Pharmacognosy, College of Pharmacy Taipei Medical University Taipei Taiwan; ^9^ Cancer Center, Wan Fang Hospital Taipei Medical University Taipei Taiwan

**Keywords:** CDK8, fragment, machine learning, molecular docking, structure‐based virtual screening

## Abstract

The identification of an effective inhibitor is an important starting step in drug development. Unfortunately, many issues such as the characterization of protein binding sites, the screening library, materials for assays, etc., make drug screening a difficult proposition. As the size of screening libraries increases, more resources will be inefficiently consumed. Thus, new strategies are needed to preprocess and focus a screening library towards a targeted protein. Herein, we report an ensemble machine learning (ML) model to generate a CDK8‐focused screening library. The ensemble model consists of six different algorithms optimized for CDK8 inhibitor classification. The models were trained using a CDK8‐specific fragment library along with molecules containing CDK8 activity. The optimized ensemble model processed a commercial library containing 1.6 million molecules. This resulted in a CDK8‐focused screening library containing 1,672 molecules, a reduction of more than 99.90%. The CDK8‐focused library was then subjected to molecular docking, and 25 candidate compounds were selected. Enzymatic assays confirmed six CDK8 inhibitors, with one compound producing an IC_50_ value of ≤100 nM. Analysis of the ensemble ML model reveals the role of the CDK8 fragment library during training. Structural analysis of molecules reveals the hit compounds to be structurally novel CDK8 inhibitors. Together, the results highlight a pipeline for curating a focused library for a specific protein target, such as CDK8.

## 1 INTRODUCTION

The preparation of screening libraries is an important starting point for a successful screening campaign. Considerations can include structural information of a protein target and favoring molecular or drug‐like properties (Follmann et al., 2019; Sadybekov & Katritch, 2023; Lyu et al., 2023). However, complications can arise as structural variations exist between targeted protein binding sites. The size of commercial screening libraries can reach into the billions, further impeding current screening methods. For these “ultra‐large” libraries, researchers have looked at computationally prioritizing molecules for synthesis and testing (Sadybekov & Katritch, 2023; Sadybekov et al., 2022). Chemical fragments, which can serve as a starting scaffold for synthesizing molecules represented within the larger chemical library, can function as starting points (Sadybekov & Katritch, 2023; Sadybekov et al., 2022; Andrianov et al., 2021). It is also conceivable that chemical fragments can aid in tailoring a screening library towards a given target. This would produce a focused library that is smaller and more manageable during the screening stage.

Molecular docking is a widely used computational tool that has been useful in identifying bioactive compounds (Sadybekov & Katritch, 2023; Lin et al., 2022; Hsu et al., 2011). Nevertheless, molecular docking can render a high number of false positives (Sadybekov & Katritch, 2023). Researchers have included several techniques that focus on a specific protein of interest, such as pharmacological interactions between the targeted protein and ligand, which can improve the scoring function of molecular docking (Lin et al., 2022; Hsu et al., 2011). Molecular docking has also proven useful in combining molecular fragment “hits” to build a larger molecule through a fragment‐growing approach (Andrianov et al., 2021; Kaplan et al., 2022). While ultra‐large screening libraries offer more molecules to explore the chemical space for a given protein target, molecular docking may produce an increased number of false positives during the initial structure‐based virtual screening (SBVS) campaign (Sadybekov & Katritch, 2023; Lyu et al., 2023; Sadybekov et al., 2022). As a result, new strategies and tools are needed to be able to efficiently screen large compound libraries.

Machine learning (ML) has become an indispensable tool in computer‐aided drug discovery. ML models can offer effective predictions of protein structures or classification of small molecule activity for a given target (Sadybekov et al., 2022; Jumper et al., 2021). They can also be used to preprocess chemical libraries towards a specific protein target (Coley, 2021). ML algorithms, such as random forest (RF), K‐nearest neighbor (KNN), logistic regression (LR), and neural networks, have proven to be important toolkits for virtual screening (Lien et al., 2023; Grimberg et al., 2022; Burki, 2020). Their uses have been promising, with several drug candidates identified or generated from ML models currently undergoing clinical evaluation (Burki, 2020). Nevertheless, several caveats should be considered. Potential bias in ML models can arise due to the size of the imbalanced training set or the type of ML algorithm used (Xu et al., 2022). This can lead to the identification of me‐too compounds that are structurally similar to previously identified drugs (Aronson & Green, 2020; Zhavoronkov et al., 2019). An ensemble model, which combines different ML algorithms, could increase the predictive prowess of ML programs and avoid biases that may arise during screening (Afolabi et al., 2018; Yu et al., 2022; Jiménez‐Luna et al., 2020). Thus, ML can be effective in creating targeted‐focused screening libraries.

Increased cyclin‐dependent kinase 8 (CDK8) expression has been found in several cancers, such as colorectal, hematological, breast, and prostate cancer (Philip et al., 2018; Chen et al., 2017). CDK8 is an important regulator of transcription and forms part of a transcription mediator complex. Overexpression of CDK8 can lead to increased transcription of nuclear factor κ‐light‐chain‐enhancer of activated B cells (NFκB) (Chen et al., 2017). Aberrant NFκB expression is implicated in the increased production of tumor‐promoting proinflammatory cytokines (Philip et al., 2018; Chen et al., 2017). These results have made CDK8 an important therapeutic target. However, an effective CDK8 inhibitor has not been developed. Identifying additional compounds could improve future design strategies for the development of novel CDK8 therapeutics.

In this study, an ensemble ML model was developed to curate a focused screening library targeting CDK8. Compounds with anti‐cancer properties targeting CDK8 have been identified using chemical fragments (Al‐Jarf et al., 2021; Rodrigues et al., 2021). Matching compounds to fragments of interest can also improve targeted ligand prediction (Zhou et al., 2021). To that end, a CDK8 fragment library was generated and used as additional features for an ensemble ML model. The ensemble model was able to reduce a library containing 1.6 million compounds by 99.90%, thereby curating a “focused” CDK8 screening library. The remaining compounds were molecularly docked to identify favorable interactions and to aid in final compound selection. Enzymatic testing revealed structurally novel CDK8 inhibitors identified from the presented screening protocol. Together, our study demonstrates how an ML model can be utilized to generate a targeted screening library to yield an effective SBVS campaign.

## 2 RESULTS

### 2.1 Project workflow

**FIGURE 1:**
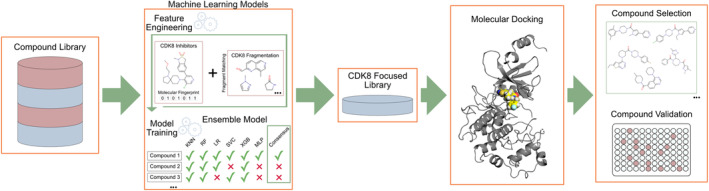
Workflow of the presented study. The ensemble ML model was generated and optimized to predict potential CDK8 inhibitors. Features were engineered from a CDK8 fragment library and molecules with known CDK8 bioactivity. The ML model reduced the parent compound library by 99.90%, resulting in a focused CDK8 screening library. The focused library was then subjected to molecular docking. Finally, candidate compounds were selected and validated for CDK8 inhibitory activity.

The workflow of the presented study is shown in Figure 1. An ensemble ML model was generated to classify CDK8 activity of query compounds. Features engineered for the ML models included molecular fingerprints of molecules with known CDK8 bioactivity as well as compound matches to a CDK8 fragment library. The CDK8 fragment library was generated using the molecular substructure miner (MoSS) algorithm (Borgelt et al., 2005). Datasets were subjected to a 90/10 split for training and testing, respectively. Combining multiple algorithms has proven useful in the identification of bioactive small molecules (Grimberg et al., 2022; Lindley et al., 2023). In this study, a query molecule must be positively selected by all six models before being placed in the CDK8‐focused library. The optimized ensemble model processed a compound library of 1.6 million compounds and reduced the parent library by 99.90%, leaving a focused library consisting of 1672 compounds. Next, the CDK8‐focused library was subjected to a SBVS campaign. Compounds were ranked based on their docking score and visually inspected for selection and testing.

### 2.2 Model analysis

**FIGURE 2:**
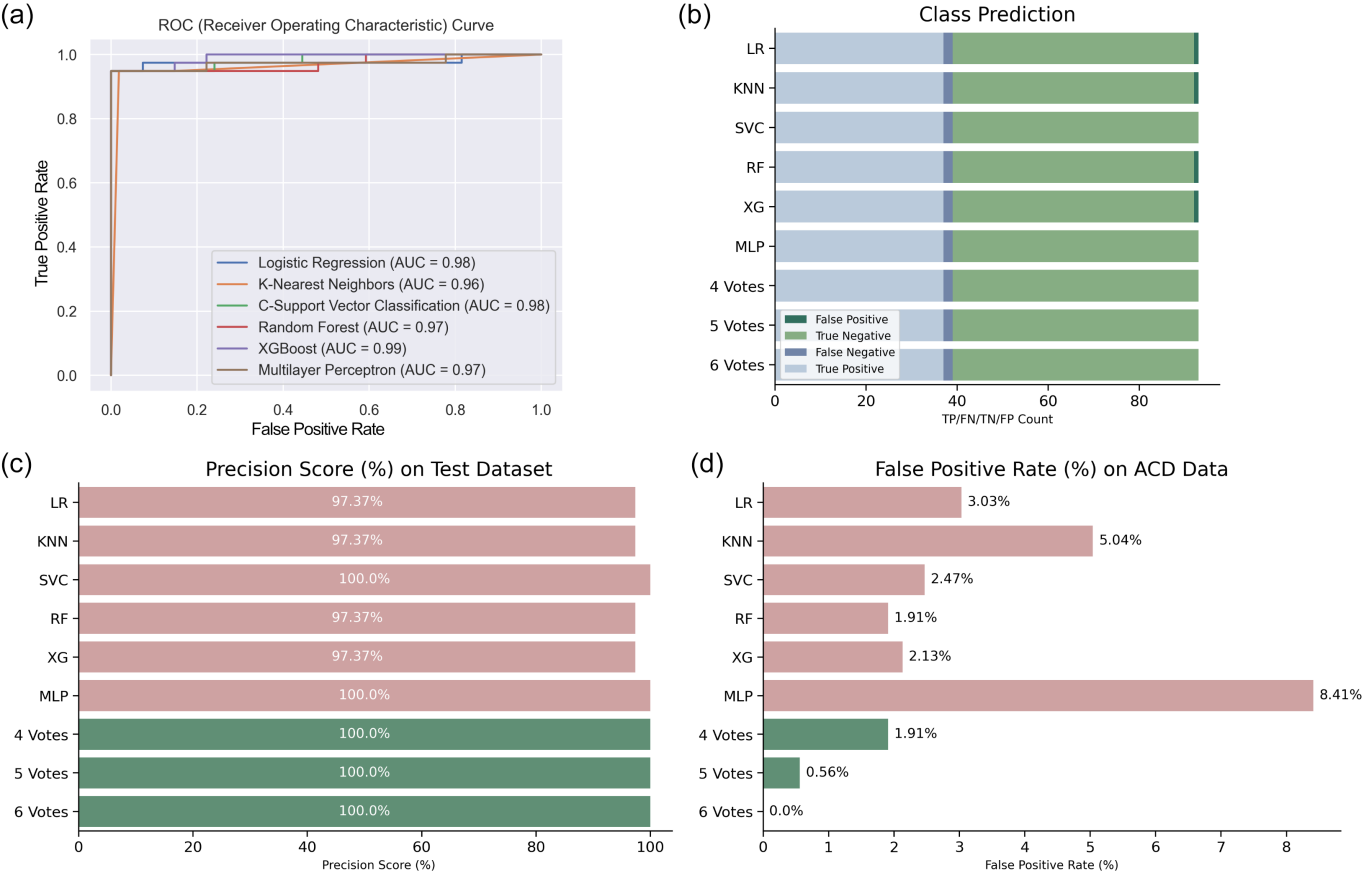
Model evaluations. (a) The area under the curve (AUC) for the indicated models shows favorable performance for CDK8 inhibitor classification. (b, c) Similar performance for models was achieved when classifying molecules. The models were tuned for greater precision. (d) The models were tested against the ACD 990 dataset, which was separated from model training and test data. Individual models showed varied performance in classification. When ensemble voting was applied, that is, whether a query compound is classified as active by a set number of models, false positives were reduced. In particular, the strict passing criteria of six models reduced the false positive rate of 0.0%. KNN, K‐nearest neighbors; LR, logistic regression; MLP, multilayer perceptron; RF, random forest; SVC, C‐support vector classification; XG, XGBoost.

Six ML classification models—LR, KNN, c‐support vector classification (SVC), RF, XGBoost, and multilayer perceptron (MLP)—were generated. Model analysis showed favorable performance, with an area under the ROC curve (AUC) score of 0.96 or higher (Figure 2a). The CDK8 dataset was imbalanced, with more molecules labeled as inactive for the training/test dataset. However, the models showed favorable classification performance and accuracy (Figure 2b). As the goal of this study was to identify potential CDK8 inhibitors, models were tuned for greater precision. This metric would indicate how often a given model is correct when classifying a molecule. High precision scores were generated for each model (Figure 2c). Further, the models showed favorable precision–recall (PR) curves and average precision (AP) scores (≥0.95), indicating suitable model performance for this study (Figure S1).

A common problem with establishing an effective ML model is data leakage. Data leakage is when information from the test data is introduced during model training (Kaufman et al., 2012). To gauge if model performance was impacted by data leakage, the models were tested on 990 randomly selected molecules from the available chemical directory (ACD) (Hsu et al., 2011). Crucially, these molecules were not part of the test set and are structures that should display no CDK8 bioactivity. The models displayed varying false positive rates, with the MLP model displaying the highest false positive rate on the ACD dataset (Figure 2d). While their performance on the ACD dataset appears favorable, they also highlight the advantages and disadvantages of the individual ML models. To compensate, we combined the models to generate a consensus “vote” per query molecule. In other words, a query molecule must be labeled as a CDK8 inhibitor by a set number of ML models for placement into a CDK8‐focused library. The ensemble model requiring a query molecule selected by four ML models produced a false positive rate of 1.91% on the ACD dataset. This was further reduced to 0% when a strict six‐vote criteria were used (Figure 2d). While the six‐vote criteria continued to produce a small number of false negatives in the testing dataset, its precision score remains higher than the individual ML models (Figure 2b,c). Importantly, against the ACD dataset, the six‐vote ensemble model produced zero false positives. Thus, the six‐vote ensemble model fits our preference for higher precision and would be used to curate molecules for a CDK8‐focused library.

### 2.3 Identification of feature importance

**FIGURE 3:**
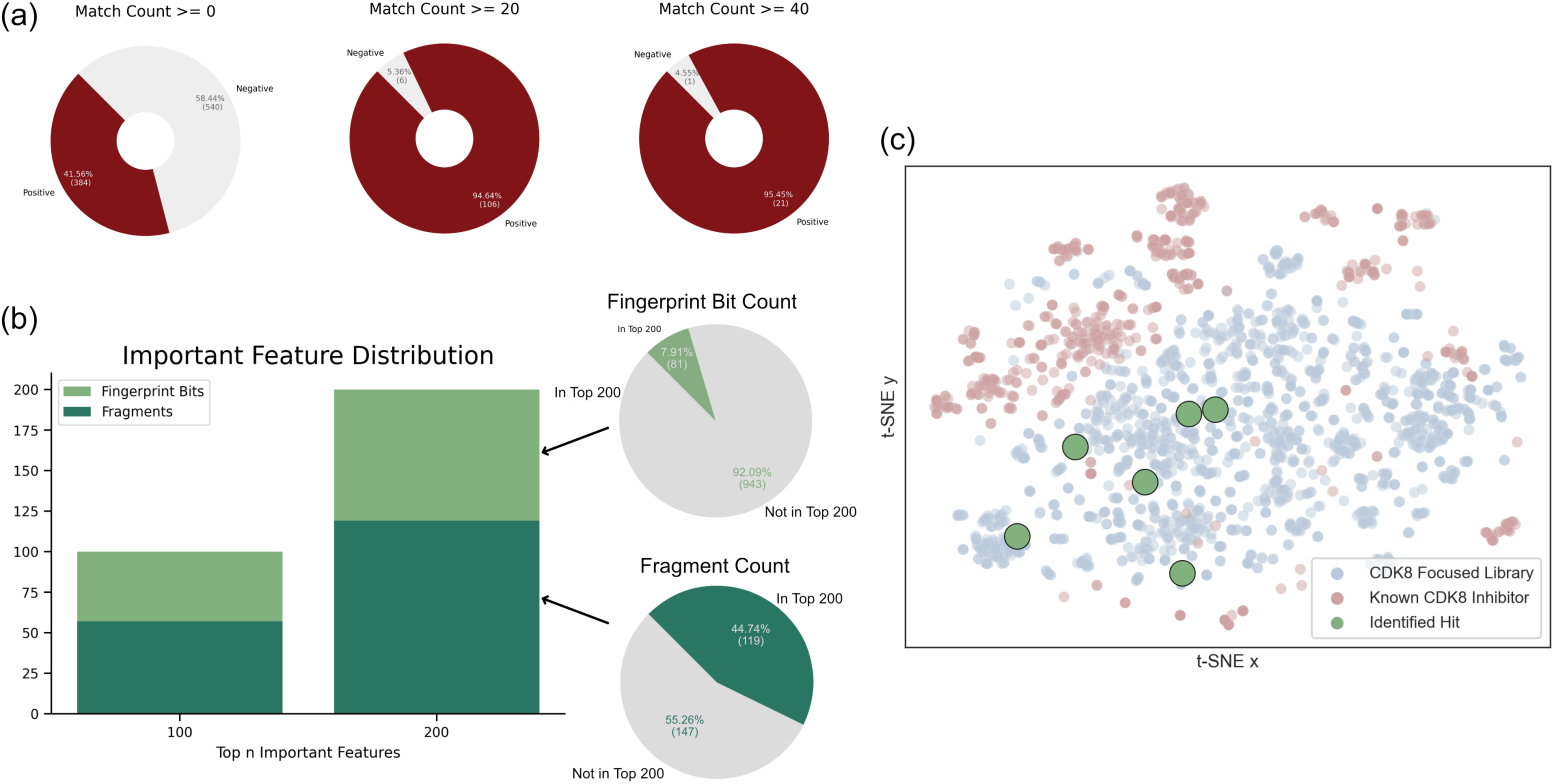
Analysis of feature importance. Model features included molecular fingerprints (1024 bits) and matches to a CDK8 fragment library. (a) Compounds lacking features with the CDK8 fragment library were likely to be labeled as inactive. (b) Important features were identified from the XGBoost model. The top 100 features showed fragments to be of high importance and continued when analyzing the top 200 features. Insert shows the number of molecular fingerprint bits or fragments that can be found in the top 200 features. (c) The curated CDK8‐focused library is diverse when compared to known CDK8 inhibitors.

We further analyzed the CDK8 fragment library and how it may influence the ML models. The features for the ML models totaled 1291 columns. Compounds were translated into 1024 circular fingerprints, making up the bulk of the features. A CDK8 fragment library was generated using the MoSS algorithm and contained 266 fragments. A similarity score was calculated between each compound and fragment, and a column with the total fragment counts for each compound was tabulated. For the ensemble ML model, compounds matching the CDK8 fragments had a greater tendency to be labeled as active (Figure 3a). In particular, the ratio of active labeled compounds increased when matching with 20 or more fragments.

Of the ML models in this study, XGBoost offers the ability to identify and analyze important model features (Boldini et al., 2023; Chen & Guestrin, 2016). CDK8 fragments made a little over half of the top 100 features for the XGBoost model (Figure S2). This was further pronounced when the importance features were increased to 200. Of the available 266 CDK8 fragments, 119 fragments were found in the top 200 features, in contrast to fingerprint bits, which only featured 81 of the 1024 fingerprints (Figure 3b).

A screening library is most useful if it contains a diverse chemical space for a given protein target (Sadybekov & Katritch, 2023; Coley, 2021). The model training may have an increased bias towards known CDK8 structures. However, when comparing the CDK8‐focused library to known CDK8 inhibitors, we found little overlap between the two (Figure 3c). Importantly, the hit compounds in this study were not clustered near groups of the known CDK8 inhibitors (Figure 3c). Together, the analysis shows the ensemble model can curate a structurally diverse CDK8‐focused library for further screening.

### 2.4 Selected compounds show CDK8 inhibitory activity

Next, the CDK8‐focused library was subjected to a SBVS campaign to identify potential CDK8 inhibitors. The re‐docking of the co‐crystal ligand confirmed favorable docking protocols for the study (Figure S3). Compounds were molecularly docked into the CDK8 binding site. Protein‐ligand interactions were generated for each ligand pose. Compounds missing hydrogen bonds to the CDK8 hinge loop were removed (Sharma & Gupta, 2022). The remaining compounds were clustered, and final selections were made based on compound poses and availability. A total of 25 candidates were selected for testing. Two compounds, T479‐0984 and G887‐0711, displayed inhibitory activity greater than 90% at 10 μM, indicating greater potency (Table 1). While seven compounds showed CDK8 inhibitory activity >50%, compounds exhibiting ≥70% were selected for further testing (Figure S4). Dose–response curves were generated for the top six compounds (Figure S5). T479‐0984 was confirmed as the most potent CDK8 inhibitor, producing an IC_50_ value of 99.9 nM, while G887‐0711 had an IC_50_ value of 1161.3 nM. These results confirm six CDK8 inhibitors identified from the CDK8‐focused library.

**TABLE 1:** Activity assays of hit compounds.

Compound	Inhibitory activity (10 μM)	IC_50_
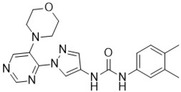 T479‐0984	99.3%	99.9 nM
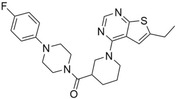 G887‐0711	91%	1161.3 nM
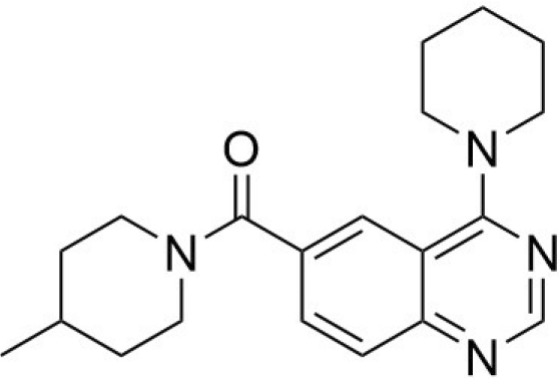 M628‐0340	77%	1924 nM
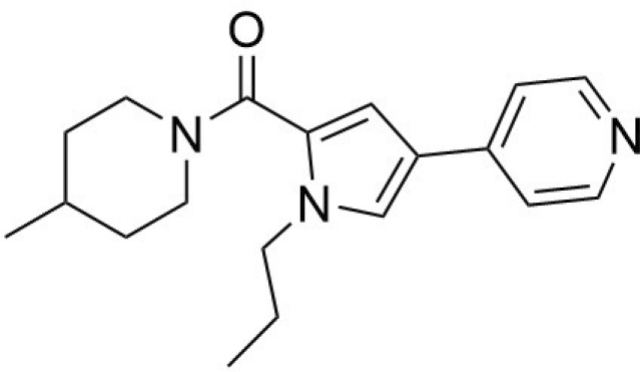 P076‐1448	73%	2262 nM
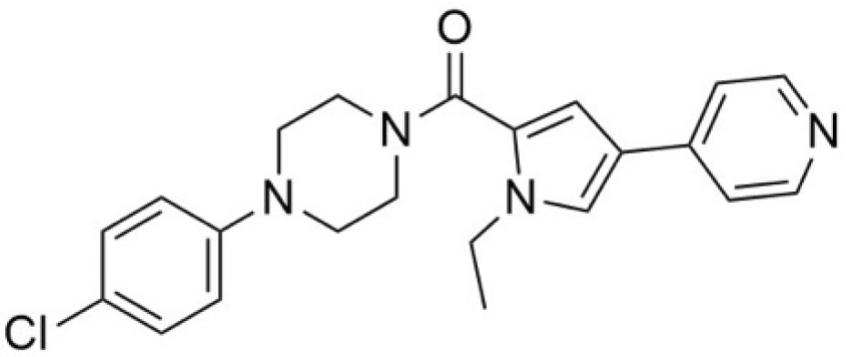 P076‐1220	71%	4442 nM
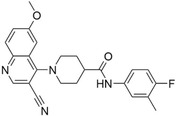 L483‐0192	71%	1837 nM

### 2.5 Structural analysis of identified inhibitors

**FIGURE 4:**
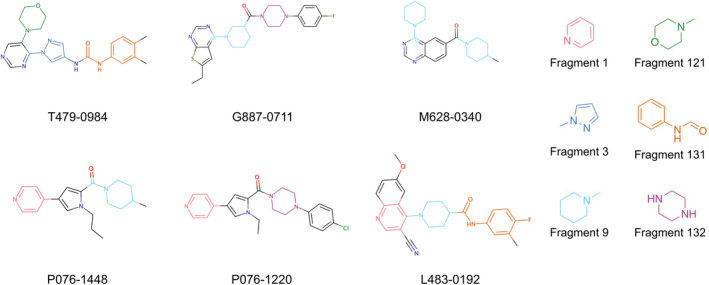
Identified inhibitors show matches to the CDK8 fragment library. The compounds and their matching fragments are shown. Representative fragments from the fragment library are highlighted in corresponding colors.

**FIGURE 5:**
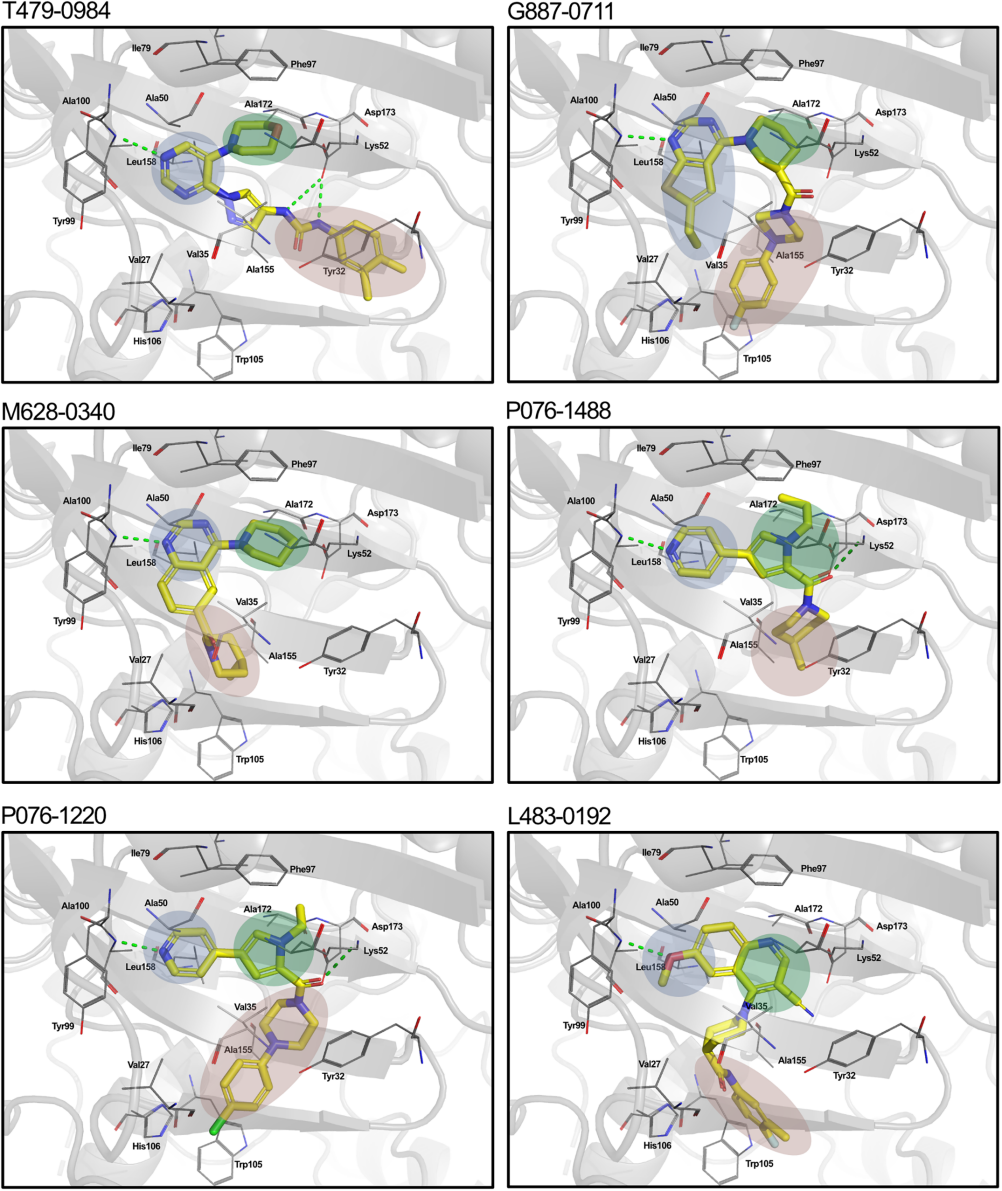
Docking poses of CDK8 inhibitors. The CDK8 inhibitors in this study show favorable occupation of the CDK8 binding site. In addition, all molecules have at least one hydrogen bond with the hinge loop (green dashes). Compounds are represented in yellow. The CDK8 binding site residues are represented in gray and labeled. The hinge region, back pocket, and solvent region are highlighted in blue, green, and red, respectively.

To better understand the identified inhibitors and their relation to the ensemble ML model, additional structural analysis of the identified CDK8 inhibitors was performed. As expected, the six compounds matched with structures within the CDK8 fragment library. The representative fragments occupy areas that may be conducive to interactions with the CDK8 binding site, such as the hinge binding, the back pocket, and the solvent region (Figure 4). Importantly, all compounds form a hydrogen bond to the CDK8 hinge loop, an important interaction observed with many kinase inhibitors targeting the kinase ATP binding site (Sharma & Gupta, 2022). In this case, the compounds generated a hydrogen bond with the hinge residue A100 (Figure 5). However, different hydrophobic interactions differ between the compounds, which can be attributed to their heterocyclic rings. Comparing protein–ligand interactions to T479‐0984 highlights binding differences that may lead to lower potency (Figure S6).

The most potent CDK8 inhibitor, T479‐094, contains a pyrimidine ring that occupies the hinge binding region. Fragment 3 and Fragment 121 flank the pyrimidine ring, with the latter extending towards the DFG loop in the back pocket of the binding site. Fragment 3 generated a van der Waals interaction with residue A50, which was not present in the other CDK8 inhibitors (Figure S6). In this study, the CDK8 structure contained the DFG loop in the “in” position, indicating an active kinase structure (Attwood et al., 2021). T479‐094 generates additional hydrogen bonds with residue D173 of the DFG loop and the nitrogens on the urea linker that can be found with the matching Fragment 131 and occupy the solvent region (Figure 5). Fragment 131 also supported van der Waals and hydrophobic interactions with residue K153 and T31. A pi‐cation interaction between the aromatic ring and K153 was also observed (Figure S6).

Both G887‐0711 and M628‐0340 contain two fused heterocyclic rings. The hinge binding region for these molecules does not match with representative fragments observed in Figure 4. G887‐0711 is the longer molecule of the two and curves towards the back pocket. In contrast, M628‐0340 is shorter and contains two fragments, which can be matched with Fragment 9, and is “forked” from its fused heterocyclic ring (Figure 5). Nevertheless, both inhibitors generate similar hydrophobic interactions, which were observed with residues A50, I79, A100, and L158. The position of the residues would coincidentally correspond to areas favorable for the ATP adenine ring (Lin et al., 2022). The spatial positioning of G887‐0711 and M628‐0340 further facilitates hydrophobic interactions with residues K52, F97, and A172 in the back pocket and residues H106 and A155 near the solvent region.

Both P076‐1448 and P076‐1220 can be considered structural analogs to each other, and both contain a carbonyl oxygen that facilitates an additional hydrogen bond to residue K52 (Figure 5). This residue is an important contributor to CDK8 activity by forming a salt bridge to residue E66. Importantly, P076‐1448 and P076‐1220 contain different fragments that may contribute to their slight difference in potency (Figure 4). P076‐1220 is a longer compound with a piperazine fragment connected to a benzene ring in the solvent region. As a result, P076‐1220 may not bind as potently as its more potent analog P076‐1448. This slight difference would present an area for future optimization.

Finally, L483‐0192 produced the most divergent docking pose of the CDK8 inhibitors identified. L483‐0192 appeared to better “hug” the periphery of the ATP binding site (Figure 5). Its terminal aromatic ring occupied the solvent region and would facilitate hydrophobic interactions with residue W105. L483‐0192 also matched with Fragment 9, contributing to hydrophobic interactions with residue V27 and V35. L483‐0192 also produced hydrophobic interactions with residues K52, F97, H106, A155, and A172, which were also observed with G887‐0711 and M628‐0340. Partial occupation of the back pocket also occurs due to the more rigid triple‐bonded nitrogen.

Together, the six identified CDK8 inhibitors show favorable binding within the CDK8 binding site. However, interaction analysis highlighted van der Waals and hydrophobic interactions present with T479‐0984, which may correlate with its greater potency. This could provide several areas for future optimization studies.

### 2.6 Fragments can influence model selections

**FIGURE 6:**
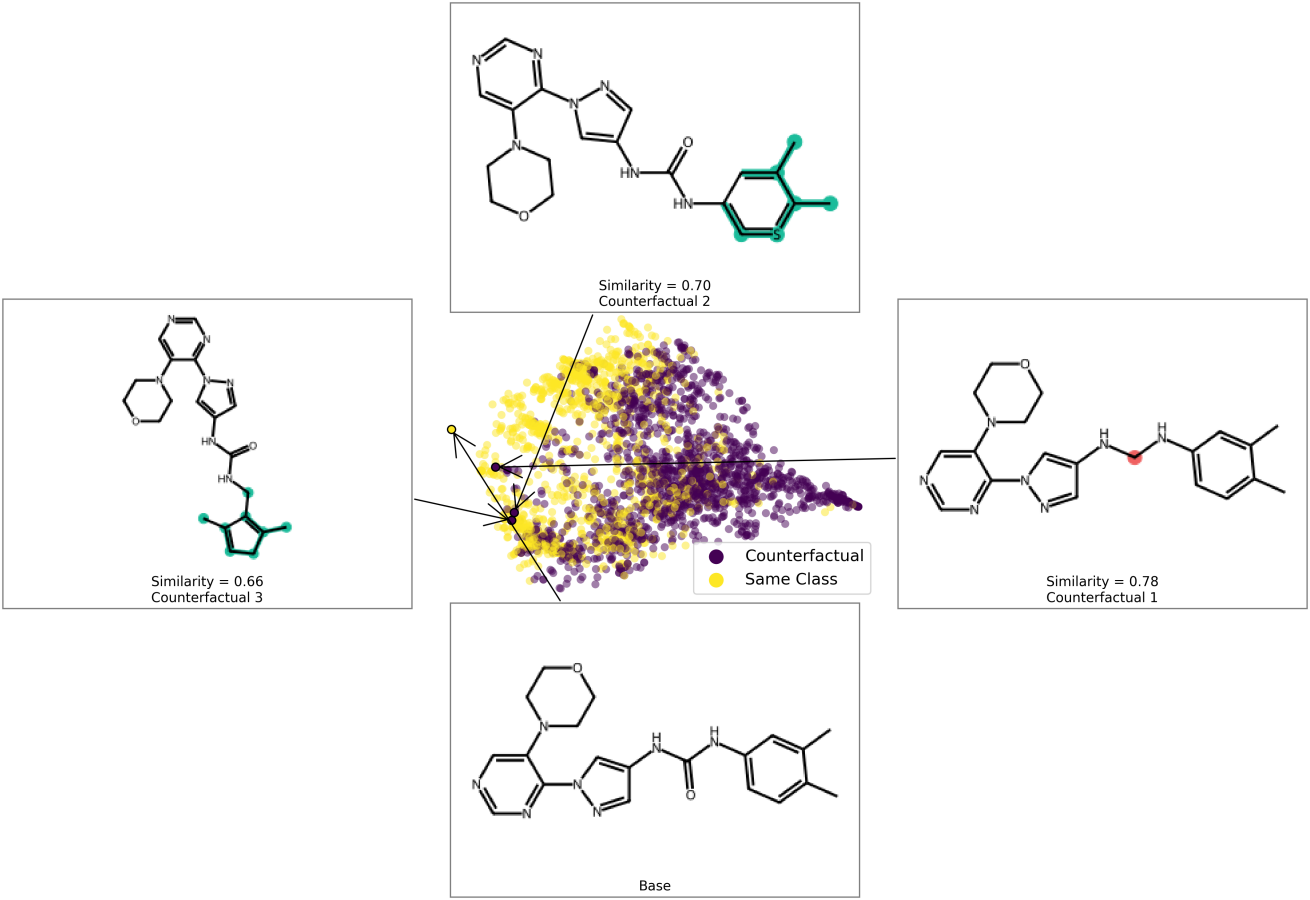
Counterfactuals denote changes to classification. Counterfactuals for the most potent CDK8 inhibitor in this study, T479‐0984, were generated from the XGBoost model. The scatterplot points to the differences between the classification of the generated molecules. Changes that require additions or removal for the base model are highlighted in green and red, respectively.

The above ML models were developed by combining molecular fingerprints from molecules with known CDK8 bioactivity and a generated CDK8 fragment library. Initial analysis showed that incorporating fragments can have an impact on the model's decision‐making (Figure S2). To better explain the model predictions, we generated counterfactuals for the identified CDK8 inhibitors. In short, a counterfactual would identify the differences in a molecule that would change its classification (Wellawatte et al., 2022). The most potent inhibitor, T479‐0984, showed changes to its terminal ring corresponding to CDK8 Fragment 131 (Figure 4). Changes that provide negative classification include a 5‐carbon ring or the change of the methyl group positions (Figure 6). The removal of the carbonyl oxygen can also contribute to a negative classification. This is consistent with the other identified CDK8 inhibitors, with many changes corresponding to areas of their matched fragments (Figure S7). Taken together, the incorporation of targeted fragments can aid in the generation of a focused screening library.

### 2.7 Structural comparisons to known CDK8 inhibitors

**FIGURE 7:**
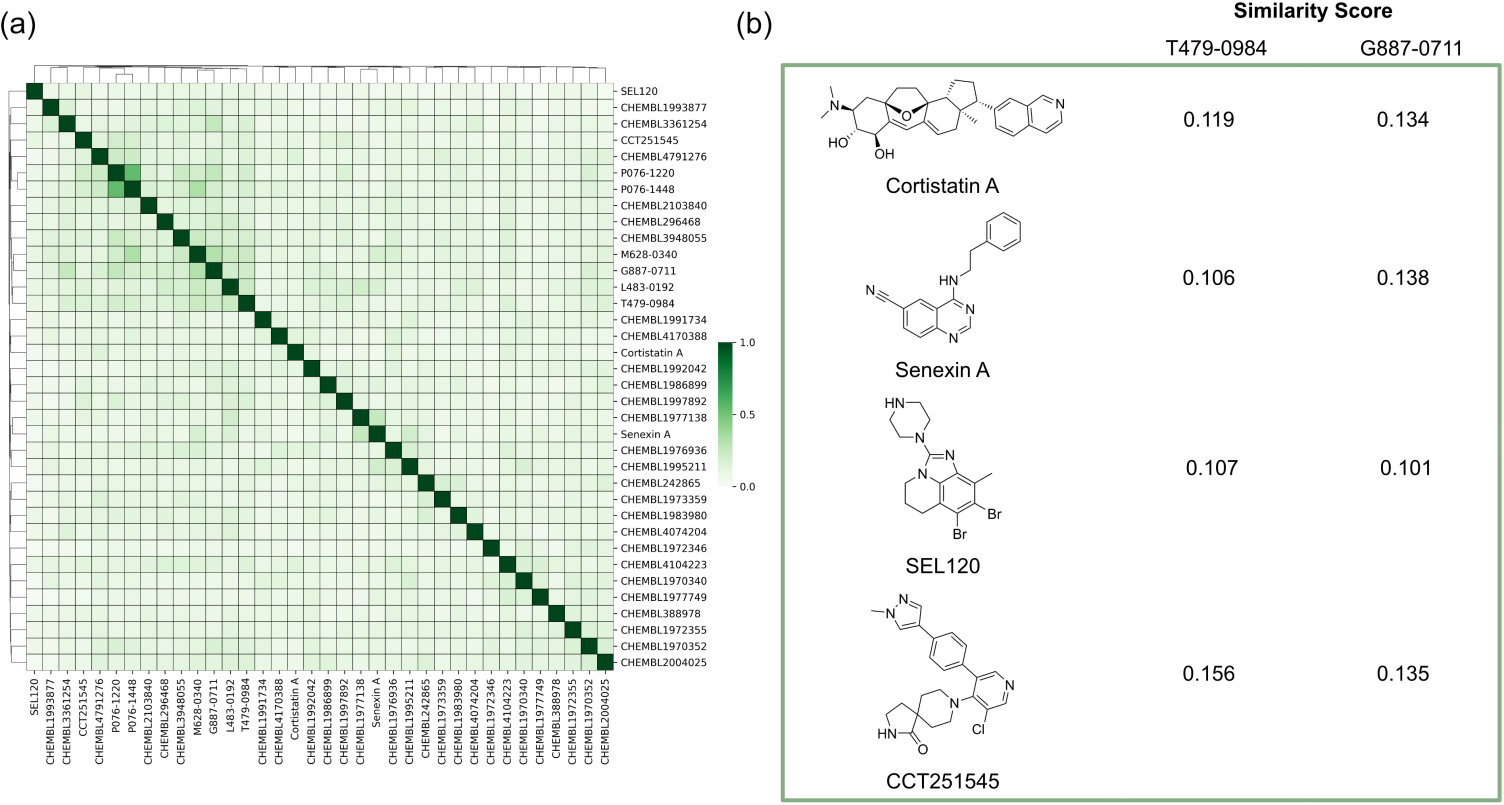
Similarity matrix of CDK8 inhibitors. (a) The identified CDK8 inhibitors and 30 diverse CDK8 inhibitors from the ChEMBL database were converted into ECFP, and their Tanimoto Scores were calculated and displayed as a heatmap. (b) Similarity scores of indicated CDK8 inhibitors compared to inhibitors T479‐0984 and G887‐0711.

An additional aim for many screening platforms is to identify inhibitors with novel structures. To compare structures, a similarity matrix was generated for the CDK8 inhibitors identified in this study with known CDK8 inhibitors. The CDK8 inhibitors were sourced from the ChEMBL database, and 30 diverse structures were selected. Overall, the identified CDK8 inhibitors did not exhibit a Tanimoto score greater than 0.300 with the known CDK8 inhibitors, suggesting structurally distinct compounds were identified from the screening campaign (Figure 7a). This coincided with the diversity of the CDK8‐focused library (Figure 3c). Inhibitors P076‐1448 and P076‐1220 were found to be most similar to each other, which is to be expected as they appear to be analogs to each other (Table 1). Well‐known CDK8 inhibitors include Cortistatin A, Senexin A, SEL120, and CCT251545 (Yu et al., 2021; Wu et al., 2021). The most potent inhibitors from our screening campaign include T479‐0984 and G887‐0711, which show low similarity scores to the aforementioned CDK8 inhibitors (Figure 7b). The six identified inhibitors expand the chemical space of CDK8 inhibitors. Importantly, they could serve as important starting points for developing therapeutic CDK8 inhibitors.

## 3 DISCUSSION

Virtual compound libraries, such as Enamine, WuXi, and CHEMriya by Otava, have reached 10 billion or more molecules (Lyu et al., 2023; Kaplan et al., 2022). Larger libraries allow probing a target protein with different chemical structures. Unfortunately, processing the entire library is not feasible with traditional high‐throughput methods. For example, high‐throughput molecular docking has been an important tool for virtually screening large datasets. However, brute docking a library containing 10^10^ molecules can take 3000 years on a single CPU (Sadybekov & Katritch, 2023). Faced with these issues, new strategies and tools are needed to more efficiently sort and filter molecules. One method is the creation of a smaller target‐focused library from the larger library. ML can be trained on molecules targeting a specific protein to generate a focused screening library that can then be subjected to more rigorous screening criteria.

Target‐focused libraries have an important role in drug discovery. An effective focused library should have a large number of compounds with great structural diversity (Harris et al., 2011). Traditional considerations for library preparation include the removal of molecules containing PAINS structures and breaking the Lipinski Rule of Five or scoring molecules for druglikeness (Lin et al., 2022). The use of fragments is another consideration. Fragments have been widely used as starting points for screening (Andrianov et al., 2021; Zhou et al., 2021). Researchers have also incorporated fragments into ML models that predict chemical fragments for lead optimization studies (Green et al., 2021). Chemical fragments can be used to alert researchers of structures that can lead to unwanted activity (Al‐Jarf et al., 2021; Rodrigues et al., 2021). These alerts would be useful in preparing a focused screening library and allow models to be tuned towards relevant chemical features that can lead to positive outcomes. Here, we utilized fragments generated from known CDK8 inhibitors as additional features in ML models.

In the realm of machine learning, many different algorithms exist. Model selection depends on a variety of factors, such as the problem to be solved and the optimization of the selected model (Lindley et al., 2023; Bergstra et al., 2013). In this study, we created an ensemble ML model based on six different ML models. Models were selected for ease of use and wide support (Chen & Guestrin, 2016; Pedregosa et al., 2011). ML models in this study include—LR, KNN, SVC, RF, XGBoost, and MLP. The LR model is a linear regression model, which seeks to fit a linear function to a given dataset to approximate classification labels (Lindley et al., 2023; Schapin et al., 2023). Support vectors are kernel‐based models and can have improved fit compared to linear models (Afolabi et al., 2018; Schapin et al., 2023). Similarity‐based models, such as KNN, identify similarities within a given data point and apply a weighted average, termed as ‘*k*’, before making a final prediction. Increased datasets can impact KNN's effectiveness, as the algorithm cannot identify complex correlations, which can result in an underfit model (Schapin et al., 2023). Two tree‐based models, RF and XGB, were used in this study. RF models include ensemble decision tree models fitted in parallel. XGB models improve upon decision tree models by fitting each tree sequentially (Chen & Guestrin, 2016). Finally, the MLP model is a type of neural network that is meant to mimic the structures of biological neurons, where models contain weighted layers for calculations (Lindley et al., 2023). Many models can suffer from overfitting. For example, LR models can overfit high‐dimensional datasets, while support vectors can have issues when features are shared between classes (Lindley et al., 2023; Schapin et al., 2023). Combining different ML models to tackle the same problem can increase predictive capabilities compared to single models (Afolabi et al., 2018; Yu et al., 2022; Lindley et al., 2023). However, ensemble models may not be the most feasible solution, as computational resources will need to be scaled accordingly with the number of algorithms used.

Individually, the models showed favorable performance (Figure 2). As our goal was to develop a model for screening potential CDK8 inhibitors, our preference was for models with higher precision scores, i.e. a model with reduced false positive classifications. Again, our test data showed a high precision score for the models (Figure 2c). Our precision–recall curves for all models featured an AP of 0.95 or greater (Figure S1). An additional test on model precision was performed from 990 chemical structures randomly selected from the ACD (Hsu et al., 2011). The structures from the ACD dataset were not observed to be ATP memetics. As a result, we expected a negative classification for all compounds in the ACD set. Importantly, compounds from the ACD set were not introduced during the training/test dataset, providing a test that would potentially avoid data leakage issues. With the ACD dataset, we observed different precision scores for the individual models (Figure 2d). Ensemble models have been shown to boost the predictive ability compared to single ML models (Afolabi et al., 2018; Yu et al., 2022). When a voting system was implemented, meaning a compound must be selected as active for a given number of models, we observed an improvement in the predictive capability. The precision was further improved as an ensemble vote system required the passing of four or more models (Figure 2c,d). These results align with the reported predictive capabilities of ensemble models when compared to singular models (Lindley et al., 2023). Currently, an effective “one‐size‐fits‐all” model is difficult to achieve. As a result, it is reasonable to add additional representative features. The incorporation of fragments from CDK8 inhibitors would aid in the identification of CDK8‐like chemical structures. An analysis of the top 200 features shows more than half of the features coming from the CDK8 fragments (Figure 3a,b). Fragments have been used to identify privileged fragments that can contribute to anti‐cancer activity (Al‐Jarf et al., 2021; Rodrigues et al., 2021). In this study, we used the MoSS tool to generate fragments that would be specific for CDK8 inhibitors. In the end, the ensemble ML model generated a CDK8‐focused library with great diversity from known CDK8 inhibitors (Figure 3b).

Despite the rise of ML tools over recent years, molecular docking continues to be an effective benchmark for finalizing selections (Cieplinski et al., 2023). Molecular docking was performed with the CDK8‐focused library and led to a selection of 25 compounds for experimental validation. Testing confirmed six compounds with CDK8 inhibitory activity (Table 1). As expected, the identified inhibitors displayed matches to the CDK8 fragment library. In fragment‐based drug design, the spatial orientation of fragments has been used to “grow” an inhibitor within a targeted pocket (Andrianov et al., 2021; Zhou et al., 2021). In contrast, we took a ligand‐based approach in the preparation of our CDK8‐focused library. The features also unveil potential preferences within the CDK8 binding site (Figures 4 and 5). Interestingly, the exmol package (Wellawatte et al., 2022), which can elucidate molecular properties for a given model prediction, showed that changes to areas corresponding to the matched fragments can change the classification of the hit compounds (Figure 6). ML models are also susceptible to me‐too selection depending on the training and optimization (Coley, 2021; Zhavoronkov et al., 2019; Lindley et al., 2023). When compared to 30 randomly and structurally distinct CDK8 inhibitors, the identified inhibitors in this study were found to be distinct (Figure 7). The CDK8‐focused screening library was also found to encompass a different space when compared to known CDK8 inhibitors (Figure 3c). Based on the dataset in this study, the CDK8‐focused screening library was structurally diverse to known CDK8 inhibitors. Taken together, the binding pose shows that the identified inhibitors can occupy the CDK8 binding site, making them interesting candidates for further optimization studies.

In this study, a screening pipeline that incorporated both machine learning and molecular docking is presented. The preparation of a screening library is a crucial step for successful screening campaigns. A CDK8‐focused library was prepared using an ensemble ML model. Importantly, this model utilized known CDK8 inhibitors as well as a CDK8 fragment library to classify molecules with features resembling CDK8 inhibitors. This produced a focused library containing 1672 molecules. The CDK8‐focused library was further screened through molecular docking, yielding 25 selections. When tested, we identified six compounds with CDK8 inhibitory activity. The structurally diverse structures from the CDK8‐focused library allowed for the identification of novel CDK8 inhibitors. The identification of the six CDK8 inhibitors will function as additional structures of study for the development of new CDK8 therapeutics.

## 4 METHODS

### 4.1 Compound datasets

Molecules with CDK8 bioactivity data were downloaded from the PubChem database (Kim, 2016). Duplicates were removed, and the molecules with the most potent activity (*K*_d_, EC_50_, or IC_50_) were retained. Active or inactive status was determined with a cutoff of less than or greater than 10,000 nM, respectively. The CDK8 dataset was used to train the model. For testing precision, a dataset containing 990 compounds was randomly selected from the ACD (Hsu et al., 2011). The screening library (1.6 million compounds) was obtained from ChemDiv (https://www.chemdiv.com). Compounds were preprocessed by removing compounds containing PAINS structures or with a qualitative estimate of drug‐likeness (QED) score of ≤0.25 (Baell & Holloway, 2010; Bickerton et al., 2012).

### 4.2 Molecular substructure mining

The CDK8 fragment library was generated using the Molecular Substructure Miner node (Borgelt et al., 2005) within the open‐source analytics platform Konstanz Information Miner (KNIME) (Berthold et al., 2007). Ring mining was selected to avoid the generation of fragments with partial rings. The dataset was mined, and fragments were retained if they appeared in 1% in the active class (minimum support), but no greater than 20% in the inactive class (maximum support). Fragments appearing more than 20% in the inactive class are assumed to increase the chance of being labeled as “inactive”, and thus were removed from the CDK8 fragment library.

### 4.3 Feature engineering

**FIGURE 8:**
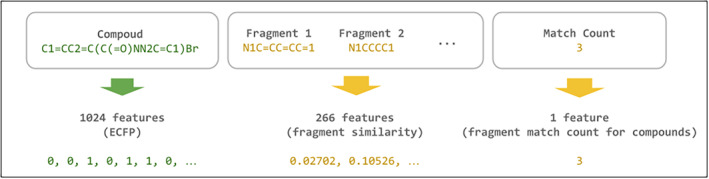
Diagram of ML model features. Molecule inputs were given as SMILES strings. The inputs were converted into 1024 ECFP bits. Compounds were matched to a CDK8 fragment library, and a final column containing the number of matches was tabulated.

A diagram of ML features can be found in Figure 8. Compound SMILES strings were used as inputs and converted into 1024 ECFP bits using the RDKit Python package (Landrum, 2013). Additionally, each molecule was compared to a fragment generated from the CDK8 fragment library as mentioned above. A Tanimoto similarity score was calculated for each molecule‐fragment pairing, producing an additional 266 features for each compound. Finally, a feature containing fragment matches between compounds and the fragment library was tabulated. Together, this produced a total of 1291 features for the ML models.

### 4.4 Machine learning models

The machine learning models were generated using Python 3.9. The Scikit‐learn (Pedregosa et al., 2011) module was used to generate the following models: RF, LR, KNN, and SVC. Two additional models were generated using the XGBoost (Chen & Guestrin, 2016) and MLP (https://keras.io) modules. To increase the model dataset, experimental results from our previous CDK8 screening campaign (Lin et al., 2022) were combined with the CDK8 dataset prepared above. Models were tuned using the Hyperopt (Bergstra et al., 2013) module. A total of 300 trials were generated for each model with a 10‐fold cross‐validation on the dataset. The hyperparameters from the best performing trial were selected for the ensemble model. The models in this study were assessed by their receiver operating characteristic (ROC) curve (Bradley, 1997). Model precision was tested on 990 randomly selected molecules from ACD. The molecules were held separate and not introduced during model training and only used for model optimization. The precision for the model was calculated as the following:

$$\text{precision}=\frac{\mathrm{TP}}{\mathrm{TP}+\mathrm{FP}}$$

where TP equals true positive, and FP equals false positive for a given model.

### 4.5 Molecular docking

The CDK8 protein structure (PDB ID: 5HBH) was downloaded from the Protein Data Bank. Molecular docking was performed using Glide (Friesner et al., 2004). The docking grid was centered on the co‐crystal ligand (PDB ligand ID: 5Y7). The protein structure was prepared by adding hydrogen atoms and associated charges, and water molecules were removed. The docking protocol was verified by re‐docking the crystal structure and comparing the resulting docking pose to the crystal pose (Figure S3). Compounds for docking were prepared by generating their 3D structures and protonated. Docking results were ranked by their docking score. Poses lacking hydrogen bonds to the hinge loop were removed. The remaining compounds were clustered, and their docking poses were visually inspected before making the final selection. Protein–compound interactions were generated using the Python module ProLIF (Bouysset & Fiorucci, 2021).

### 4.6 Kinase activity assay

Selected compounds were tested using the Thermo Fisher Scientific SelectScreen kinase profiling service (www.thermofisher.com/selectscreen). The LanthaScreen kinase activity assay was used. In short, a reaction between fluorescein‐labeled substrate and ATP is performed in a container. The reaction is ceased with the addition of EDTA. A terbium‐labeled antibody is added to detect phosphorylated fluorescein‐labeled substrate. A ratio between the acceptor and donor signal is an indicator of the amount of phosphorylated substrate present in the reaction, thereby indicating kinase activity.

### 4.7 Structural similarity analysis

Known CDK8 inhibitors were obtained from the ChEMBL database (Mendez et al., 2019). Structures were clustered into a set of 30 clusters, and representative compounds were selected. A Tanimoto similarity score was calculated for each compound and active CDK8 inhibitor in this study using RDKit. The final heatmap was generated using the Seaborn module (Waskom, 2021).

## AUTHOR CONTRIBUTIONS

**Kai‐Cheng Hsu:** Conceptualization; funding acquisition; investigation; supervision; methodology; project administration; validation; writing – review and editing. **Tony Eight Lin:** Conceptualization; data curation; investigation; methodology; software; formal analysis; visualization; writing – original draft; writing – review and editing. **Dyan Yen:** Investigation; software; visualization. **Wei‐Chun HuangFu:** Investigation; supervision. **Yi‐Wen Wu:** Investigation; formal analysis; validation. **Jui‐Yi Hsu:** Resources. **Shih‐Chung Yen:** Resources. **Tzu‐Ying Sung:** Software. **Jui‐Hua Hsieh:** Software. **Shiow‐Lin Pan:** Investigation; supervision. **Chia‐Ron Yang:** Investigation; supervision. **Wei‐Jan Huang:** Supervision; investigation.

## Supporting information


**FIGURE S1.** Model precision–recall curves. The individual models contained a precision–recall score ≥ 0.95.
**FIGURE S2.** Model feature importance. The feature importance was obtained from the XGBoost model. Shown are the top 100 features of the model.
**FIGURE S3.** Docking validation. The co‐crystal ligand (ligand ID: 5Y7) was re‐docked into the CDK8 crystal structure. Superimposing the docking pose with the crystal pose generated an RMSD of 0.55 Å.
**FIGURE S4.** Compounds not selected for further testing. The results of compounds with inhibitory activity <70%. Compounds were tested at 10 μM.
**FIGURE S5.** Dose–response curves of hit compounds. Compounds were tested at 10,000, 3000, 1000, 300, and 100 nM. Assays were performed using ThermoFisher kinase assay as described in Section 4.
**FIGURE S6.** Interaction analysis of CDK8 inhibitors. Protein–ligand interactions were generated for the CDK8 inhibitors. (A) The interaction heatmap shows molecules lacking van der Waals or hydrophobic interactions compared to the more potent T479‐0984. (B) The 2D protein–ligand interaction diagram highlights where residues interact with T479‐0984.
**FIGURE S7.** Generated counterfactuals for hit CDK8 inhibitors. Counterfactuals for hit CDK8 inhibitors were generated from the XGBoost model. Many changes correspond to areas that match with fragments from the CDK8 fragment library. Additions or removal of structures are highlighted in green or red, respectively.

## Data Availability

Datasets for this project, such as the CDK8 training set, CDK8 fragment library, and the ACD990 dataset and relevant code can be found at the following link: https://github.com/TonyEightLin/CDK8-ensemble.git.
