# Supplementary material for: An ensemble machine learning model generates a focused screening library for the identification of CDK8 inhibitors
Source: Protein Sci. 2024 May 9;33(6):e5007. doi: 10.1002/pro.5007 (PMC11081523; doi:10.1002/pro.5007)
Supplement: Supplementary file 1 — FIGURE S1. Model precision–recall curves. The individual models contained a precision–recall score ≥ 0.95. FIGURE S2. Model feature importance. The feature importance was obtained from the XGBoost model. Shown are the top 100 features of the model. FIGURE S3. Docking validation. The co‐crystal ligand (ligand ID: 5Y7) was re‐docked into the CDK8 crystal structure. Superimposing the docking pose with the crystal pose generated an RMSD of 0.55 Å. FIGURE S4. Compounds not selected for further testing. The results of compounds with inhibitory activity <70%. Compounds were tested at 10 μM. FIGURE S5. Dose–response curves of hit compounds. Compounds were tested at 10,000, 3000, 1000, 300, and 100 nM. Assays were performed using ThermoFisher kinase assay as described in Section 4. FIGURE S6. Interaction analysis of CDK8 inhibitors. Protein–ligand interactions were generated for the CDK8 inhibitors. (A) The interaction heatmap shows molecules lacking van der Waals or hydrophobic interactions compared to the more potent T479‐0984. (B) The 2D protein–ligand interaction diagram highlights where residues interact with T479‐0984. FIGURE S7. Generated counterfactuals for hit CDK8 inhibitors. Counterfactuals for hit CDK8 inhibitors were generated from the XGBoost model. Many changes correspond to areas that match with fragments from the CDK8 fragment library. Additions or removal of structures are highlighted in green or red, respectively. [file PRO-33-e5007-s001.docx]

**Supplementary Figures**

**An ensemble machine learning model generates a focused screening library for the identification of CDK8 inhibitors**

Tony Eight Lin^1,2^, Dyan Yen^1^, Wei-Chun HuangFu^1,2,3^, Yi-Wen Wu^1^, Jui-Yi Hsu^1,2^, Shih-Chung Yen^4^, Tzu-Ying Sung^5^, Jui-Hua Hsieh^6^, Shiow-Lin Pan^1,2,3^, Chia-Ron Yang^7^, Wei-Jan Huang^8^,

Kai-Cheng Hsu^1,2,3,9,*^

^1^Graduate Institute of Cancer Biology and Drug Discovery, College of Medical Science and Technology, Taipei Medical University, Taipei, Taiwan

^2^Ph.D. Program for Cancer Molecular Biology and Drug Discovery, College of Medical Science and Technology, Taipei Medical University, Taipei, Taiwan

^3^TMU Research Center of Cancer Translational Medicine, Taipei Medical University, Taipei, Taiwan

^4^Warshel Institute for Computational Biology, The Chinese University of Hong Kong (Shenzhen), Shenzhen, Guangdong, People’s Republic of China

^5^Biomedical Translation Research Center, Academia Sinica, Taipei, Taiwan

^6^Division of Translational Toxicology, National Institute of Environmental Health Sciences, National Institute of Health, Durham, NC, USA

^7^School of Pharmacy, College of Medicine, National Taiwan University, Taipei, Taiwan

^8^Graduate Institute of Pharmacognosy, College of Pharmacy, Taipei Medical University, Taipei, Taiwan

^9^Cancer Center, Wan Fang Hospital, Taipei Medical University, Taipei, Taiwan

*Corresponding author

E-mail: piki@tmu.edu.tw (K.C. Hsu)


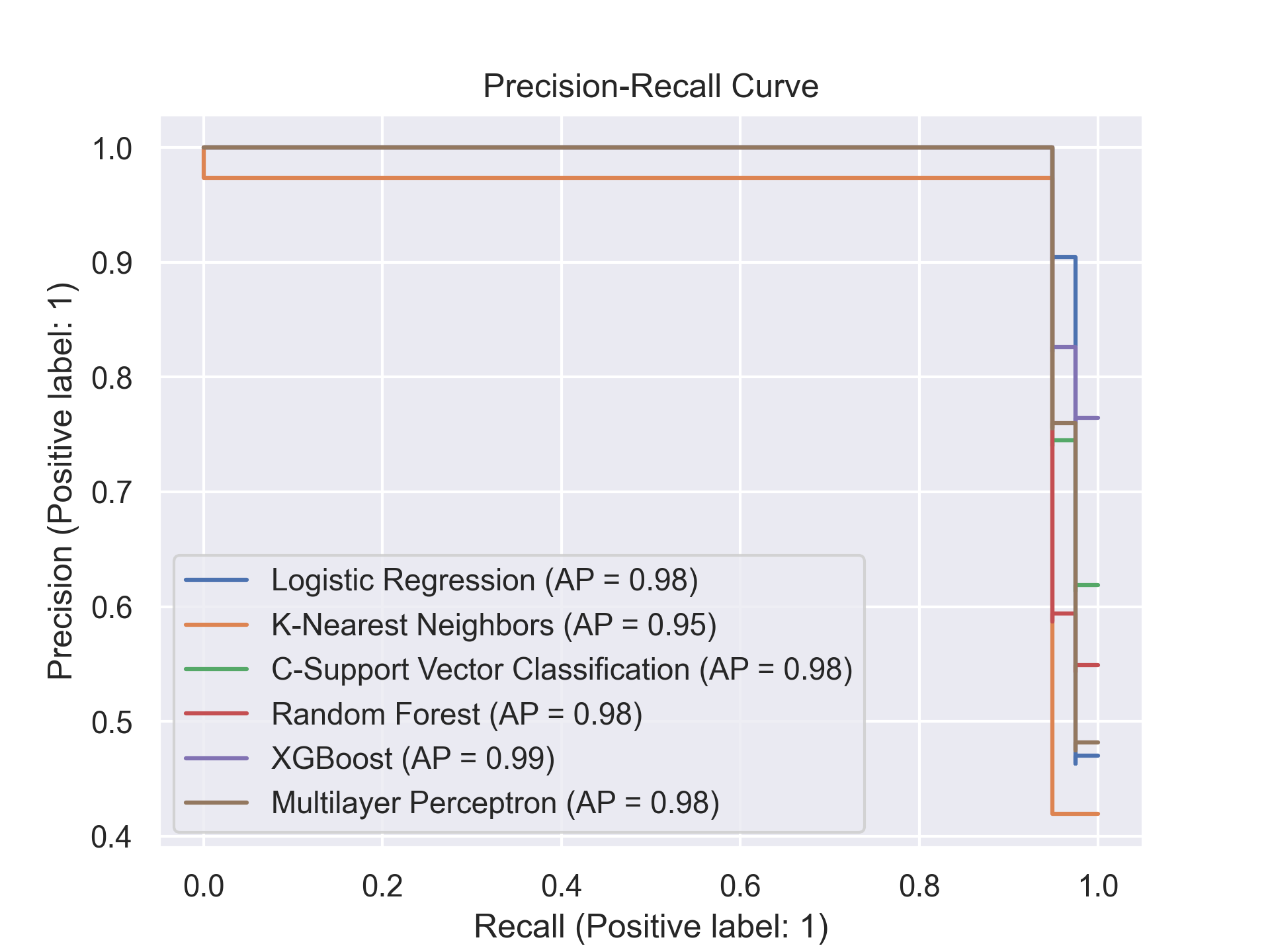


**Supplementary Figure 1.** **Model Precision-Recall Curves**. The individual models contained an average precision (AP) score ≥ 0.95.


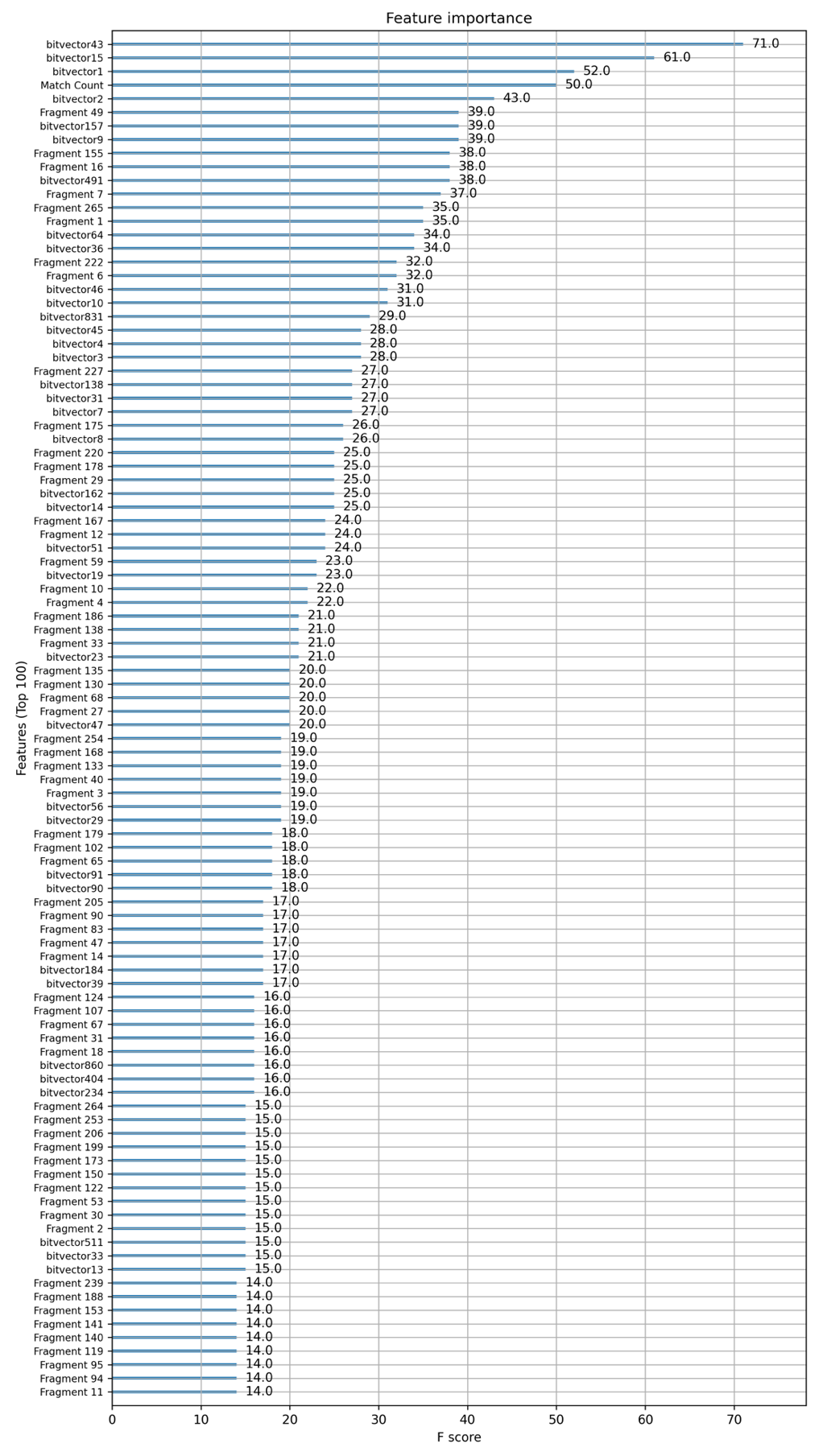


**Supplementary Figure 2. Model feature importance.** The feature importance was obtained from the XGBoost model. Shown are the top 100 features of the model.

**Supplementary Figure 3. Docking validation.** The co-crystal ligand (Ligand ID: 5Y7) was re-docked into the CDK8 crystal structure. Superimposing the docking pose with the crystal pose generated an RMSD of 0.55 Å.

| 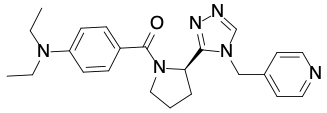  CM2843-7686  Inhibitory Activity: 61.86% | 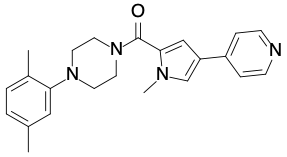  P076-0224  Inhibitory Activity: 44.00% | 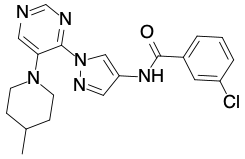  T479-0152  Inhibitory Activity: 42.34% |
| --- | --- | --- |
| 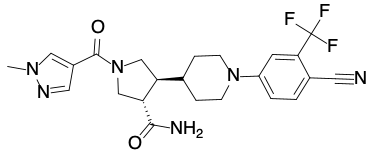  S954-0146  Inhibitory Activity: 39.33% | 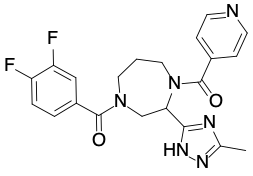  SA53-1095  Inhibitory Activity: 36.66% | 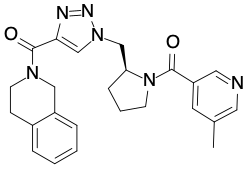  SB20-1110  Inhibitory Activity: 36.35% |
| 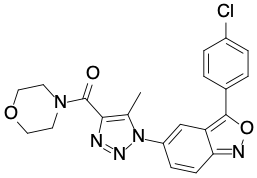  D443-1998  Inhibitory Activity: 36.25% | 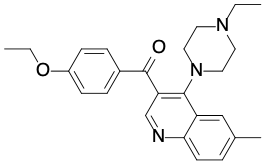  C768-1378  Inhibitory Activity: 35.17% | 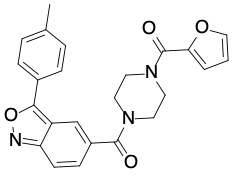  G609-0298  Inhibitory Activity: 32.84% |
| 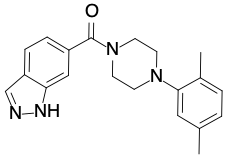  G339-0015  Inhibitory Activity: 32.02% | 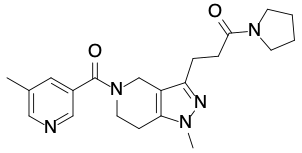  SB99-0549  Inhibitory Activity: 31.59% | 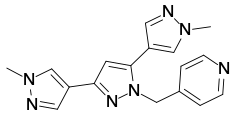  Y505-6954  Inhibitory Activity: 30.47% |
| 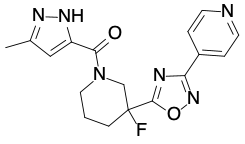  SA92-0296  Inhibitory Activity: 29.69% | 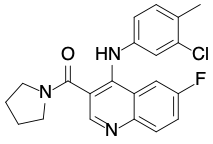  F080-0918  Inhibitory Activity: 29.15% | 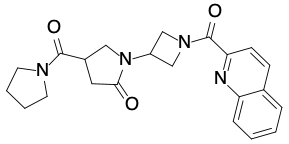  S689-0330  Inhibitory Activity: 28.02% |
| 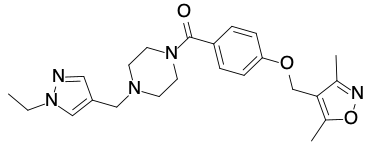  Y504-8284  Inhibitory Activity: 26.70% | 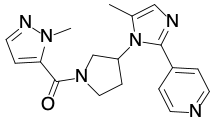  S694-1315  Inhibitory Activity: 24.52% | 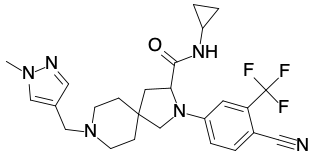  S953-5411  Inhibitory Activity: 23.99% |
| 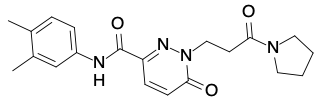  F550-3594  Inhibitory Activity: 17.31% |  |  |

**Supplementary Figure 4.** **Compounds not selected for further testing.** The results of compounds with inhibitory activity < 70%. Compounds were tested at 10 µM.

**Supplementary Figure 5. Dose-response curves of hit compounds.** Compounds were tested at 10,000, 3,000, 1,000, 300, and 100 nM. Assays were performed using ThermoFisher kinase assay as described in methods.


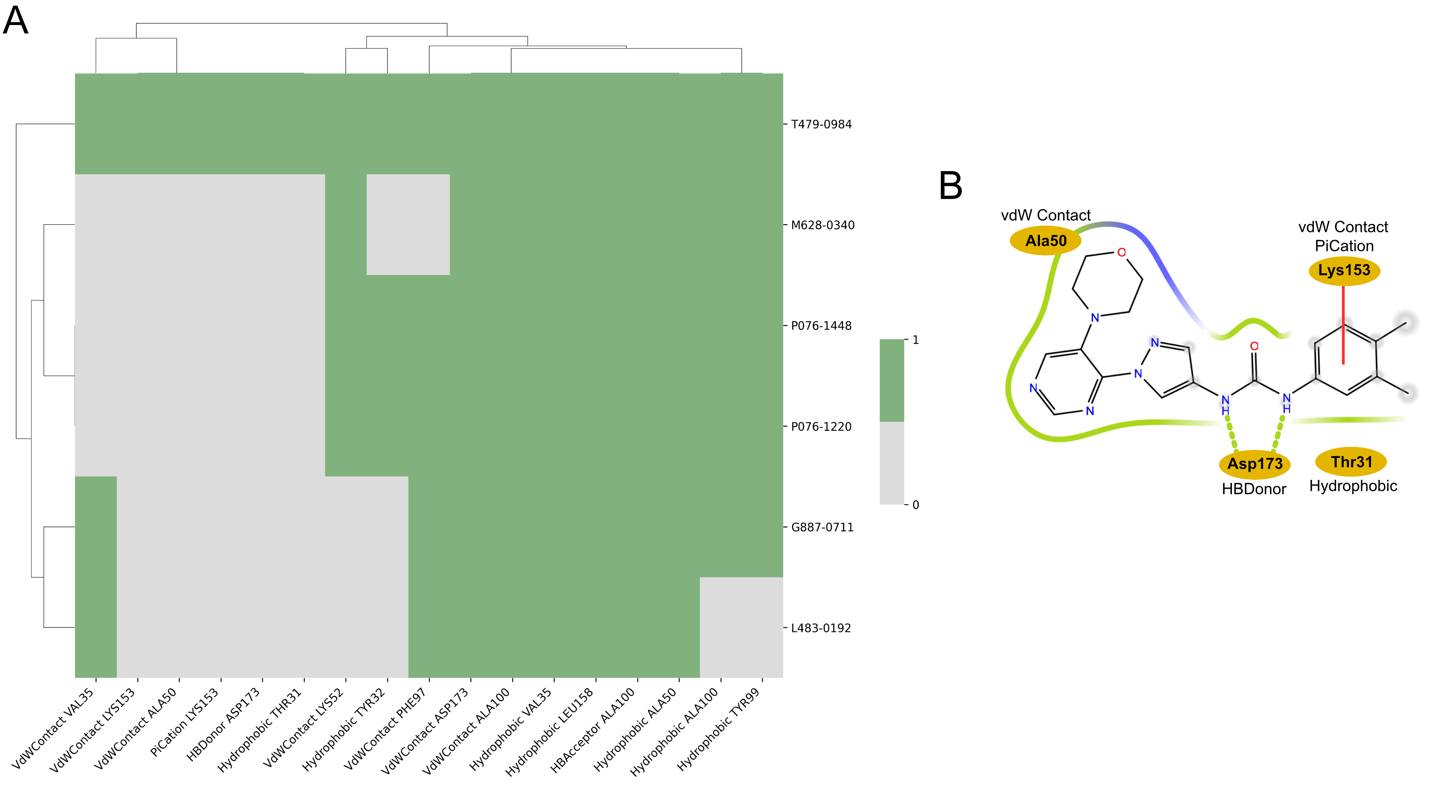


**Supplementary Figure 6. Interaction analysis of CDK8 inhibitors.** Protein-Ligand interactions were generated for the CDK8 inhibitors. (A) The interaction heatmap shows molecules lacking van der Waals or hydrophobic interactions compared to the more potent T479-0984. (B) The 2D protein-ligand interaction diagram highlights where residues interact with T479-0984.


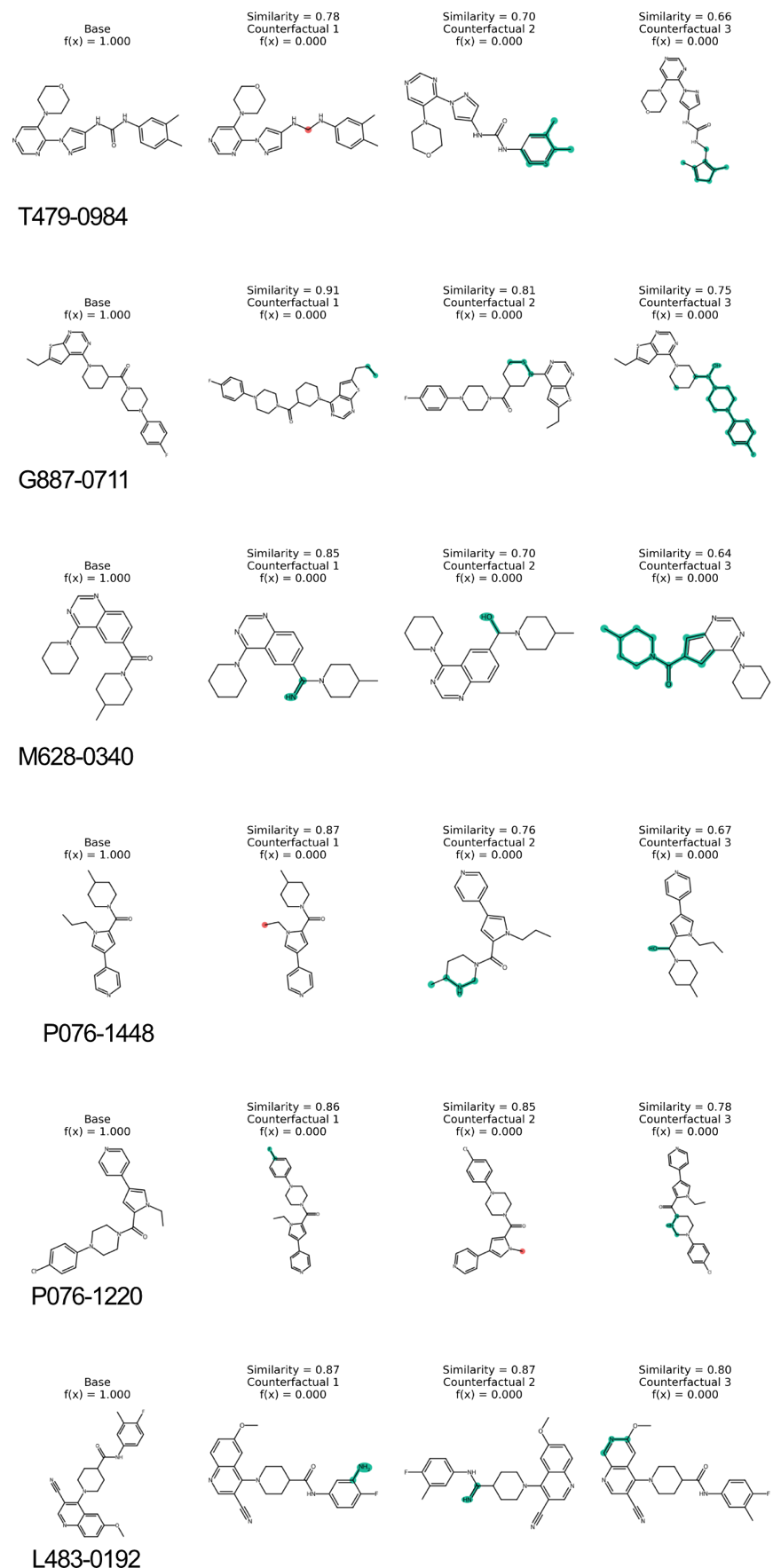


**Supplementary Figure 7. Generated counterfactuals for hit CDK8 inhibitors.** Counterfactuals for hit CDK8 inhibitors were generated from the XGBoost model. Many changes correspond to areas that match with fragments from the CDK8 fragment library. Additions or removal of structures are highlighted in green or red, respectively.
